# Draft Genome of the Marine Gammaproteobacterium *Halomonas titanicae*

**DOI:** 10.1128/genomeA.00083-13

**Published:** 2013-03-14

**Authors:** Cristina Sánchez-Porro, Rafael R. de la Haba, Norge Cruz-Hernández, Juan M. González, Cristina Reyes-Guirao, Laura Navarro-Sampedro, Modesto Carballo, Antonio Ventosa

**Affiliations:** a Department of Microbiology and Parasitology, Faculty of Pharmacy, University of Sevilla, Sevilla, Spain; b Department of Applied Physics I, University of Sevilla, Sevilla, Spain; c Institute of Natural Resources and Agrobiology, Spanish National Council for Research (IRNAS-CSIC), Sevilla, Spain; d Biology Service, CITIUS, University of Sevilla, Sevilla, Spain

## Abstract

*Halomonas titanicae* strain BH1 is a heterotrophic, aerobic marine bacterium which was isolated from rusticles of the RMS Titanic wreck. Here we report the draft genome sequence of this halophilic gammaproteobacterium.

## GENOME ANNOUNCEMENT

The marine bacterium *Halomonas titanicae* strain BH1 was isolated from a sample of rusticles, which are formed in part by a consortium of microorganisms, collected from the RMS Titanic wreck site (1). This bacterium was previously characterized as a new species of the genus *Halomonas*, which includes a large number of species isolated predominantly from marine, hypersaline, or alkaline habitats (saline lakes, salterns, salted food, etc.). Although these species are easily isolated from saline and hypersaline environments, genome data are currently available only from the type species of the genus *Halomonas*, *H. elongata* (2). *Halomonas titanicae* is a Gram-negative, heterotrophic, aerobic rod, motile by peritrichous flagella. Phylogenetically this organism belongs to the *Gammaproteobacteria* within the family *Halomonadaceae* (3, 4). This halophilic organism has a respiratory metabolism, being able to grow in media with 0.5 to 25% NaCl (optimal growth at 2 to 8% NaCl); no growth occurs in the absence of NaCl (1). Strain BH1 is the type strain of the species *H. titanicae* and has been deposited in several culture collections as ATCC BAA-1257, CECT 7585, JCM 16411, and LMG 25388.

The draft genome sequence of *Halomonas titanicae* strain BH1 was obtained using a whole-genome shotgun strategy (5) with Roche 454 pyrosequencing technology on a GS FLX titanium system (Roche Diagnostic, Branford, CT) at the Biology Service, CITIUS, University of Sevilla, Spain, consisting of single-end reads (66,683 reads, totaling 34.1 Mb) with approximately 8.8-fold coverage of the entire genome. All reads were assembled into 48 contigs (longer than 603 bp) using GS *de novo* assembler 2.3 (454 Life Sciences, Branford, CT). The coding regions were predicted using Glimmer 3.02 (6) and the functional annotation of these predicted genes was achieved with InterProScan (7). The rRNA and tRNA genes were found using RNAmmer (8) and tRNAscan (9), respectively.

The draft genome includes 5,339,792 bp with a G+C content of 55.3% and is composed of 3,314 putative protein-coding genes or open reading frames (ORFs). Furthermore, the strain BH1 contains 3 rRNA operons and a total of 58 tRNA genes. The genome of *H. titanicae* shows important gene features related to metal corrosion. For example, numerous metal-depending and -binding genes are found, including iron reductases, iron uptake regulators, ferrochelase, iron transporters, and iron-binding periplasmic protein-encoding genes. A variety of metallopeptidases are also present in this genome, pointing to a metal. Nitrate reductases are also present. As a halophilic bacterium, this genome also shows properties related to solute and ion transport, with over 16 genes putatively encoding solute-binding proteins, 18 sodium/solute symporters and transporters detected in this study, and osmolyte-related genes (i.e., ectoin synthase as an example).

### Nucleotide sequence accession numbers.

The *Halomonas titanicae* BH1 whole-genome shotgun project has been deposited at DDBJ/EMBL/GenBank under the accession number AOPO00000000. The version described in this paper is the first version, AOPO01000000.
